# Optimizing Solvent Selection and Processing Conditions to Generate High Bulk-Density, Co-Precipitated Amorphous Dispersions of Posaconazole

**DOI:** 10.3390/pharmaceutics13122017

**Published:** 2021-11-26

**Authors:** Derek Frank, Luke Schenck, Athanas Koynov, Yongchao Su, Yongjun Li, Narayan Variankaval

**Affiliations:** 1 Process Research & Development, Merck & Co., Inc., Rahway, NJ 07065, USA; luke_schenck@merck.com (L.S.); athanas.koynov@merck.com (A.K.); 2 Analytical Research & Development, Merck & Co., Inc., Rahway, NJ 07065, USA; yongchao.su@merck.com (Y.S.); yongjun.li@merck.com (Y.L.); narayan_variankaval@merck.com (N.V.)

**Keywords:** co-precipitated amorphous dispersion, posaconazole, Form III, Form γ, processing–performance relationship, solvent selection, plasticization, continuous processing, co-processed API

## Abstract

Co-precipitation is an emerging method to generate amorphous solid dispersions (ASDs), notable for its ability to enable the production of ASDs containing pharmaceuticals with thermal instability and limited solubility. As is true for spray drying and other unit operations to generate amorphous materials, changes in processing conditions during co-precipitation, such as solvent selection, can have a significant impact on the molecular and bulk powder properties of co-precipitated amorphous dispersions (cPAD). Using posaconazole as a model API, this work investigates how solvent selection can be leveraged to mitigate crystallization and maximize bulk density for precipitated amorphous dispersions. A precipitation process is developed to generate high-bulk-density amorphous dispersions. Insights from this system provide a mechanistic rationale to control the solid-state and bulk powder properties of amorphous dispersions.

## 1. Introduction

Administering active pharmaceutical ingredients (APIs) in their amorphous phase is a well-documented approach to improve the bioavailability of poorly soluble compounds [1,2]. Most often, amorphous drugs are stabilized from crystallization by molecular-level mixing with inert excipients to form a class of materials known as amorphous solid dispersions (ASDs) [3]. Such excipients, traditionally polymers, reduce crystallization propensity in the solid-state via an anti-plasticizing effect and, relative to the pure amorphous phase, can improve properties such as the dissolution rate, processability, and stability of dissolved supersaturated drug in vivo [4]. Process design is an essential component for controlling the properties of pharmaceutical materials and is especially relevant for amorphous solid dispersions [5,6]. One crucial quality attribute of ASDs is the absence of crystallinity, as crystallization during processing is common and can negate the potential bioavailability enhancement of amorphous dosage units [7,8]. As a result, there is dedicated work to develop manufacturing approaches for amorphous materials that lower crystallization risk while optimizing process efficiency and robustness [9,10,11,12]. Additionally, innovations in process design sought to improve the powder attributes of amorphous materials such as bulk density, flowability, and compressive strength [13,14,15,16]. The ideal route for generating amorphous solid dispersions controls both the solid-state and bulk powder properties of ASDs to maximize robustness and processability during manufacturing [1,17,18].

**Figure 1:**
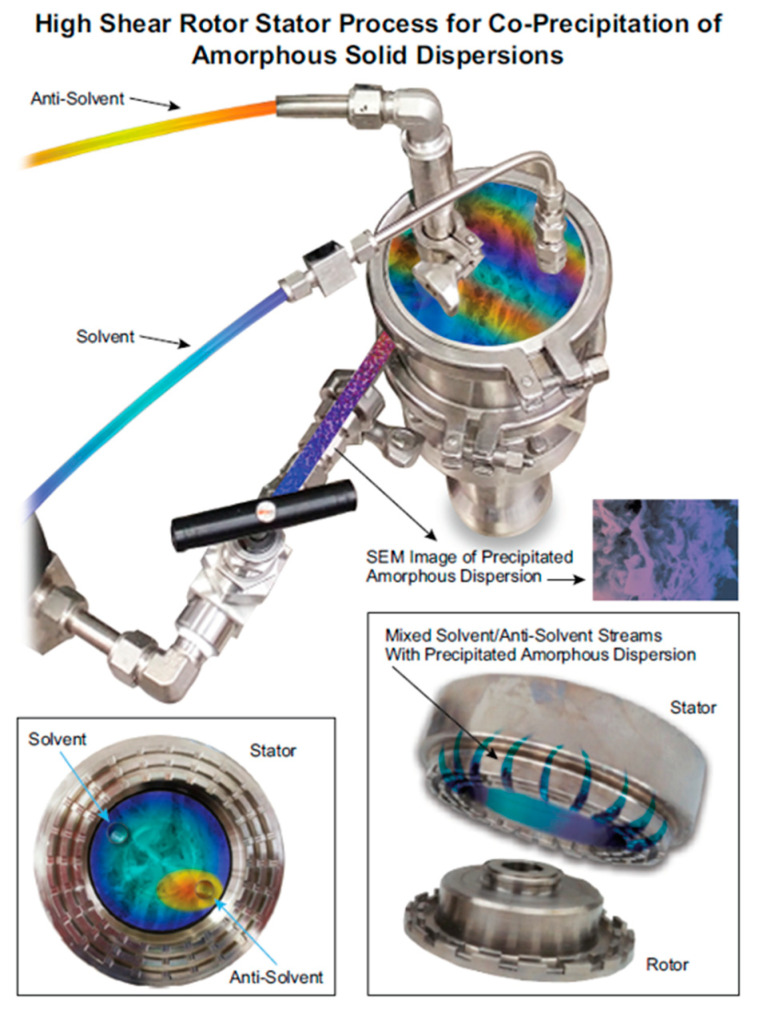
Schematic representing the co-precipitation process to generate amorphous solid dispersions. A solution of API and polymer is precipitated via rapid mixing with an anti-solvent in the rotor-stator device. Cut away highlights the stream addition and geometry of the mill tooling (Quadro HV0).

A number of processing approaches have been used to generate amorphous materials in the pharmaceutical industry, with novel technologies continuing to evolve in both academia and industry [9,19]. Of these, two manufacturing approaches have dominated: hot melt extrusion and spray drying. Hot melt extrusion of drugs and polymers is most applicable to generate amorphous dispersions for low melting-point drugs [20,21], whereas spray drying is used most broadly to manufacture amorphous solid dispersions [17]. While both of these technologies have distinct roles in the manufacturing toolkit for amorphous solid dosage forms, novel processing technologies emerged to meet the growing physicochemical complexities of the modern pharmaceutical pipeline [22] and to enable the production of amorphous dispersions containing pharmaceuticals with properties rendering them otherwise intractable by conventional approaches [17,23,24,25,26,27,28,29]. Co-precipitation is one such manufacturing approach that has garnered interest in the pharmaceutical industry for its ability to generate amorphous dispersions for APIs with high melting points and poor solubility in volatile organic solvents [3,25,30,31,32,33,34] Co-precipitated amorphous dispersions (cPAD) may also show improved mechanical properties [35] and material properties such as surface area [33] and crystallinity [3] relative to ASDs generated by more conventional means. In the co-precipitation process, API and polymer are co-dissolved in a common solvent and precipitated via reverse addition into an anti-solvent under rapid mixing (see Figure 1). Amorphous materials are achievable using this approach due to technological advances enabling the rapid mixing of miscible solvents and anti-solvents [3]. Using modern rotor-stator devices, mixing on the time scale of milliseconds brings solvent and anti-solvent into molecular contact, generating a homogeneous supersaturation field and the separation of a liquid phase (consisting of solvent and anti-solvent) from a solid amorphous phase (consisting of the drug embedded in polymer) prior to crystal nucleation and growth [36,37].

Despite ongoing research on precipitative routes for amorphous materials, the selection of processing parameters, including solvent selection, remains a primarily empirical exercise. This reality is also true for spray drying [12,38], and a growing body of literature has demonstrated how solvent selection has a substantial influence on the solid-state properties of ASDs, such as crystallinity and particle morphology [6,13,39,40,41,42]. However, a mechanistic understanding to guide solvent selection remains elusive for manufacturing technologies of amorphous materials. In this work, co-precipitated amorphous dispersions of posaconazole were prepared in a range of solvents. Following the patent literature [43,44], polymorphs of posaconazole were prepared and characterized as controls to identify crystallinity in the co-precipitated dispersions. Posaconazole was selected as a model compound because of its well-differentiated thermal signature of dispersions containing residual crystallinity. Additionally, despite work on amorphous solid dispersions and pure amorphous forms of posaconazole, there is little known on the relationship between solvent selection during manufacturing and the solid-state properties of the resulting amorphous materials [45,46,47,48,49]. It was found that posaconazole dispersions precipitated into an aqueous anti-solvent contained no residual crystallinity, as opposed to those prepared by co-precipitation into *n*-heptane. However, the material generated from aqueous anti-solvents showed poor powder properties such as a low bulk density. Informed by the mechanism of crystallization of dispersions in *n*-heptane, an approach was developed to optimize both the molecular and material properties of posaconazole cPAD. This consolidated unit operation could produce fully amorphous cPAD with a high bulk density for facile formulation and enhanced bioavailability downstream with line-of-sight to other APIs. In summary, this work offers tangible solutions to the challenging problem of solvent selection for co-precipitated dispersions and provides insights into the nature of process design to form amorphous dispersions.

## 2. Materials and Methods

### 2.1. Materials

Posaconazole was synthesized by Merck & Co. Inc., Kenilworth, NJ, USA; posaconazole is a BCS Class II triazole medication to treat fungal infections [50,51]. The polymer HPMCAS-M grade was a kind gift from ShinEtsu (Tokyo, Japan). The solvents methyl ethyl ketone (MEK), tetrahydrofuran (THF), acetone, methyl *tert*-butyl ether (MTBE), reagent alcohol, cyclohexane, and *n*-heptane were purchased from Fisher Scientific (Waltham, MA, USA).

### 2.2. Preparation

#### 2.2.1. Spray-Dried Intermediate

Amorphous posaconazole dispersions were generated by spray drying on a ProCepT 4M8-TriX Formatrix spray dryer equipped with a 0.6 mm bifluid nozzle. A solution of polymer and API (at 25% drug load) in methanol was sprayed against a stream of air flowing at 0.4 m^3^/min. The solution was fed into the atomizing nozzle at a rate of 5 mL/min and atomized using compressed air at 70 psi. The inlet and outlet temperatures were 75 and 45 °C, respectively. The spray-dried intermediate (SDI) was collected and stored at room temperature in 10 mL amber glass bottles in a desiccator.

#### 2.2.2. Melt Quenched Dispersion

Physical mixtures of posaconazole and HPMCAS-M (25% DL) were prepared by weighing components and cryomilling (SPEX CertiPrep 6750 with liquid nitrogen as coolant) at 10 Hz for five 2 min cycles, each followed by a 2 min cooldown. To make the amorphous solid dispersions, the milled API–polymer mixtures were melted at 290 °C for 20 min and then ground using a mortar and pestle. This material was amorphous by PXRD and showed the same thermal properties as amorphous dispersions of the same composition prepared by hot melt extrusion [52].

#### 2.2.3. Co-Precipitated Amorphous Dispersion

Processing conditions were selected following previous work on co-precipitation [30,33]. Posaconazole (anhydrous Form I) and HPMCAS-M were dissolved in solvent (28.5 mM API concentration, 25% drug load) and fed into the high shear zone of a Quadro HV0 wet mill in a recycle loop containing *n*-heptane (1:10 solvent:anti-solvent ratio) at −10 °C over a period of ~60 s with a peristaltic pump. Tip speeds ranged between 10–50 m/s on the Quadro, and were explored without significant changes in the solid-state properties of the resulting cPAD.

For precipitation into acidified water, posaconazole (36 mM, or ~29% DL relative to API and polymer in solution) was dissolved in solvent to account for extraction of posaconazole into anti-solvent during precipitation and processing, and used to generate dispersions at 25% DL in the solid state. Anti-solvent was cooled to 5 °C with temperatures increasing to 15 °C by completion of the precipitation.

cPAD formed from all solvent systems was isolated by filtration and dried in a vacuum oven at room temperature with a dry nitrogen sweep to remove solvent. Samples were stored in dry conditions, and protected from moisture at ambient temperatures.

#### 2.2.4. cPAD Stability in Solvent Mixtures

cPAD stability in solvent systems was determined by resuspending amorphous cPAD (precipitated from acetone into acidified water) in various solvent combinations. Approximately 7 mg/mL cPAD was agitated in solvents for at least 30 min before isolating by filtration and analyzing by mDSC.

#### 2.2.5. Posaconazole Form III

Posaconazole Form III was generated following patent procedures [44] An additional method was found to generate Form III by reverse, anti-solvent precipitation of a 28.5 mM solution of posaconazole in methyl ethyl ketone (MEK) into dry-ice-chilled *n*-heptane agitated with a T25 rotor stator device. The precipitate was isolated by filtration and dried with a nitrogen sweep.

#### 2.2.6. Posaconazole Form γ

Form γ of posaconazole was prepared following methods detailed in patent literature via conversion of Form III [44]. Form III was prepared as described above and heated in a vacuum oven at 140 °C and ~2 in Hg with a light nitrogen sweep over 3 h to facilitate conversion to Form γ. Successful conversion was verified by DSC with a single melting endotherm above 170 °C (and no solid-state thermal events associated with anhydrous Form I during heating) and unique PXRD pattern.

### 2.3. Characterization

#### 2.3.1. X-ray Powder Diffraction

PXRD data for amorphous dispersions were collected on a D2 PHASER Benchtop X-ray Powder Diffraction (Bruker) at 30 kV, 10 mA using CuKα radiation (λ = 1.54187 Ǻ) from 5° to 40° 2θ with a scan speed of 0.25 s/step and a step size of 0.02°.

PXRD data for posaconazole polymorphs were collected on a Bruker D8 Advance Powder X-ray Diffractometer (copper X-ray tube, Cu Kα = 1.54 Å, 40 kV, 40 mA, Madison, WI, USA).

#### 2.3.2. Differential Scanning Calorimetry (DSC)

Dispersions were analyzed using modulated differential scanning calorimetry using a Q2000 DSC from TA instruments in TZero hermetically sealed pans containing a pinhole. Modulation was set to ±1 °C per minute and scans were collected by ramping at 3 °C/min to above the melting point of posaconazole (250 °C).

Posaconazole crystalline phases were characterized using the same Q2000 DSC without modulation using a linear temperature ramp of 10 °C/min. The glass transition of amorphous posaconazole in Form III sample was observed using modulated DSC at a 5 °C/min scan rate with modulation set to ±1 °C per minute.

#### 2.3.3. Solid-State NMR Spectroscopy

Solid-state NMR (ssNMR) experiments were carried out using a Bruker Avance III HD 400 spectrometer (Bruker, Billerica, MA, USA) operating at proton frequencies of 399.87 MHz, respectively, in the Biopharmaceutical NMR Laboratory (BNL) at Pharmaceutical Sciences, MRL (Merck & Co., Inc., West Point, PA, USA). All experiments were conducted with a MAS frequency of 12 kHz at 294 K. All ^13^C ssNMR spectra were obtained with a Bruker 4 mm HFX MAS probe in double-resonance mode. ^13^C spectra were compared with adamantane peaks. All data were processed in Bruker TopSpin software (Bruker, Billerica, MA, USA).

#### 2.3.4. Bulk Density

Bulk density of dispersions was determined by weighing cPAD powder charged into a glass vial without agitation of the powder cake. The weight of a known volume of powder was determined to calculate bulk density, and relative bulk densities were compared by charging equal masses into scintillation vials.

## 3. Results/Discussion

**Figure 2:**
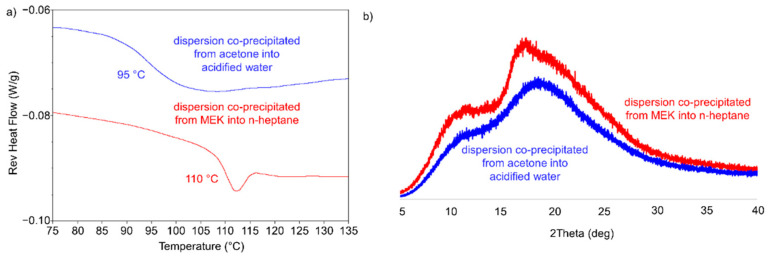
(**a**) Reversing heat flow of co-precipitated materials through their glass transitions (noted on plot) and (**b**) PXRD diffractograms for co-precipitated dispersions.

The solid-state properties of co-precipitated dispersions of posaconazole in HPMCAS-M (25% DL) depend on both the solvent and anti-solvent used for co-precipitation. Illustrated in Figure 2, two distinct phases prepared by co-precipitation from different solvents can be differentiated on the basis of their thermal properties. Co-precipitation of a posaconazole/HPMCAS-M solvent stream into acidified water generates a material that is amorphous by PXRD and has a T_g_ of 95 °C (Figure 2). This glass transition temperature is in line with that of the amorphous spray-dried intermediate or hot melt extrudate of the same composition [52] A separate, unique phase was generated at the same drug loading by precipitation from MEK using *n*-heptane as anti-solvent and was differentiated by what appeared to be an elevated glass transition temperature relative to the amorphous cPAD phase (Figure 2a). Yet, despite its elevated T_g_, which would suggest posaconazole crystallization from the amorphous phase, the material generated from *n*-heptane appeared amorphous by PXRD and did not show endothermic events at high temperature associated with the melting of crystalline posaconazole.

**Figure 3:**
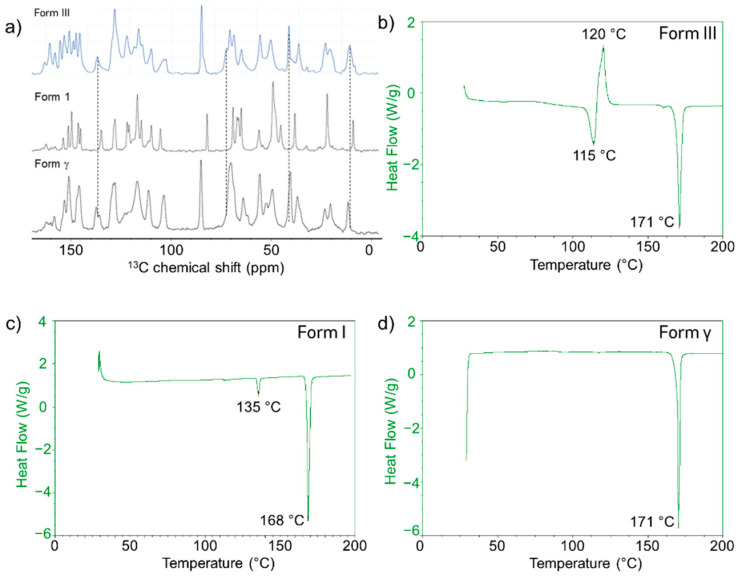
(**a**) Comparison of ^13^C-ssNMR of posaconazole forms III, I, and γ (lines drawn to highlight differences between Form III and Form γ), and differential scanning calorimetry thermograms of posaconazole: (**b**) Form III, (**c**) Form I, and (**d**) Form γ.

**Figure 4:**
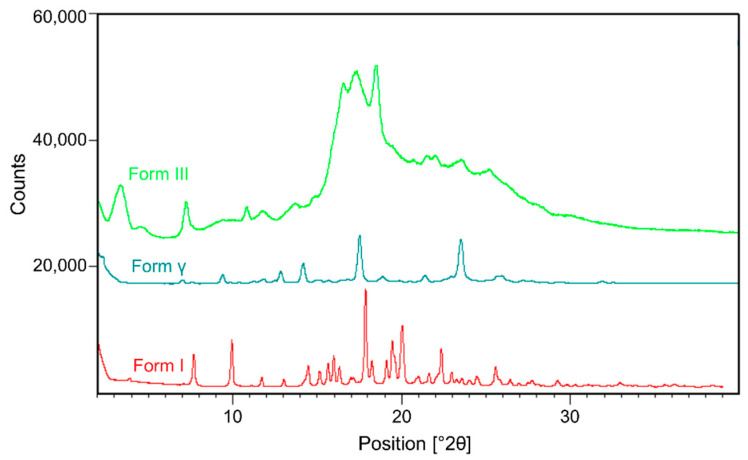
PXRD of crystalline posaconazole Forms I, γ, and III.

To understand the differences in the solid-state properties of co-precipitated posaconazole dispersions, crystalline posaconazole polymorphs were prepared and characterized. Despite the common use of posaconazole as a model pharmaceutical in amorphous dispersions [46,47,48,52,53], its crystalline phase behavior is not fully understood and still a subject of current research [45,54]. There are at least four known crystal forms of posaconazole [43,44,55]. Forms I, III and γ were isolated and compared using ^13^C-NMR spectroscopy, DSC, and PXRD, shown below in Figure 3 and Figure 4. Solid-state packing of these crystal phases can be readily differentiated using ^13^C-ssNMR spectroscopy (Figure 3a). Thermal behavior of each crystal phase is also differentiated by differential scanning calorimetry. Form III undergoes melting at 115 °C and recrystallization to Form γ (Figure 3b), Form I exhibits an endotherm at 135 °C and melts at 168 °C (Figure 3c), and Form γ melts at roughly 171 °C (Figure 3d). Finally, powder X-ray diffraction reveals differences in the lattice arrangement of these crystal phases (Figure 4). The broad amorphous hump in the diffractogram of Form III originates from contamination by amorphous posaconazole, which can be detected orthogonally using mDSC by the T_g_ of the pure amorphous drug at 60 °C (see Supporting Information) [56].

**Figure 5:**
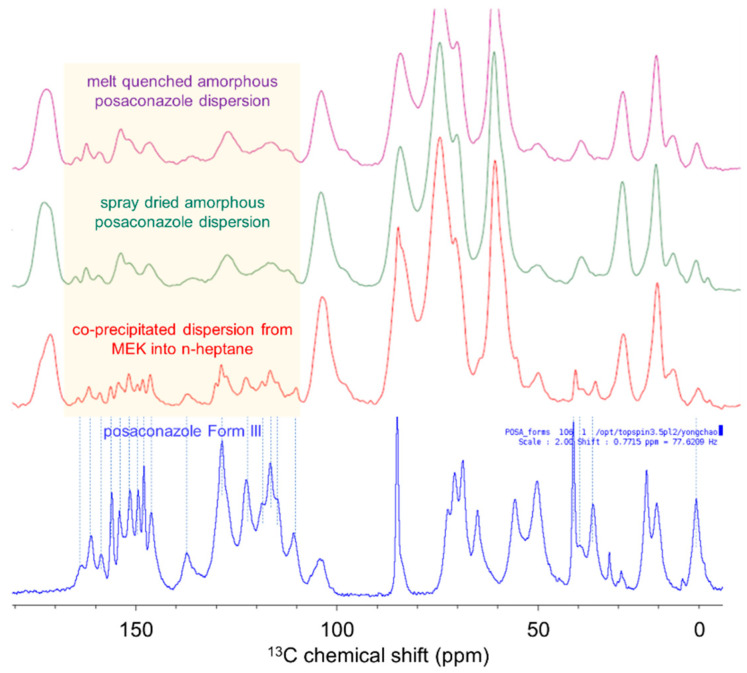
ssNMR spectra of posaconazole dispersion co-precipitated from MEK into *n*-heptane compared with fully amorphous materials and crystalline posaconazole (Form III).

Following the characterization of the posaconazole polymorphs, ^13^C-ssNMR spectroscopy was performed on the co-precipitated dispersion generated from MEK into *n*-heptane to identify the origin of its elevated glass transition temperature. Shown in Figure 5, the precipitate contains spectral features by ssNMR identical to those for crystalline Form III, in contrast with the amorphous dispersions with glass transition temperatures at 95 °C. Although the *n*-heptane dispersion does not show bulk crystallinity by PXRD, there appears to be a broad peak at ~17° 2θ (Figure 2b), which may correspond with weakly diffracting crystallites of Form III posaconazole. Additionally, although there is no melt endotherm at elevated temperatures, thermal analysis of the dispersion generated from *n*-heptane shows an endotherm concurrent with the glass transition at 110 °C (Figure 2a). This endotherm may result from the melting of Form III contaminate in the amorphous dispersion. Melting point depression of Form III in the presence of HPMCAS could lower the melting point to ~110 °C. Upon melting, posaconazole likely induces the glass-to-rubber transition of the stabilizing polymer as it dissolves into and plasticizes the solid state. Heating the *n*-heptane dispersion through the endotherm at 110 °C results in the complete dissolution of posaconazole into the stabilizing polymer, producing an amorphous material with a T_g_ at 95 °C (see Supporting Information). Overall, the combination of Form III by ssNMR and a T_g_ plasticized above the anticipated 95 °C indicate the presence of Form III in the co-precipitated dispersion generated from *n*-heptane, although additional analytical techniques to elucidate crystallite size in the dispersion are outside the scope of this work. In general, the occurrence of phase separation and crystallization are common in amorphous dispersions during manufacturing and administration [57,58]. What is intriguing about this system is that co-precipitated dispersions generated in 0.001 N HCl do not show any evidence of crystallinity, contrasting with dispersions generated in *n*-heptane. The clear thermal signature differentiating each material provides an opportunity to probe how processing conditions can impact residual crystallinity in amorphous dispersions of posaconazole.

The relationship between the solvent combination used in co-precipitation and residual crystallinity of dispersions was explored for a range of solvents and anti-solvents. Summarized in Table 1, co-precipitation of posaconazole and HPMCAS into *n*-heptane or cyclohexane anti-solvents produced a material with crystallinity, regardless of the solvent used to dissolve the API and polymer. Both miscible and immiscible solvent combinations were investigated, and the resulting precipitates were analyzed by mDSC to confirm the presence of crystalline posaconazole in the solid state (see Supporting Information for DSC thermograms). The use of binary solvent systems such as mixtures of THF and MEK failed to eliminate the presence of crystalline posaconazole, and neither did decreasing the ratio of solvent to anti-solvent for the co-precipitation (ratios as high as 1:50 solvent:anti-solvent by volume were explored). Although co-precipitation into nonpolar anti-solvents such as *n*-heptane is a successful approach for generating cPAD for other APIs [32], and is required to generate dispersions stabilized by non-enteric polymers such as VA64 [33], such processing conditions led to residual crystallinity for posaconazole/HPMCAS-M dispersions.

In contrast, posaconazole ASDs without residual crystallinity were generated by co-precipitation using acidified water (0.001 N HCl) as an anti-solvent (Table 1). Co-precipitation from solvents, including acetone and ethanol/water, into acidified water produced amorphous material with T_g_ in line with that of the fully amorphous SDI or HME material, which did not show Form III posaconazole by ssNMR spectroscopy. Although co-precipitation using THF, a more hydrophobic solvent [59], into acidified water generated a phase-separated dispersion with two T_g_, no observable crystallinity in the precipitate was detected by DSC. These data show that, for dispersions of posaconazole, the formation of crystalline API during co-precipitation correlates with the polarity of the anti-solvent. To understand this further, activity coefficients of a common solvent (acetone) were computed in Dynochem using the non-random, two-liquid model (NRTL) in both water and *n*-heptane. Against expectations, acetone activity was lower in heptane than in water, indicating that the difference in the behavior of the precipitations into nonpolar and aqueous anti-solvents does not stem from differential activity coefficients of the solvent (see Supporting Information). Instead, we hypothesize that the differences in properties for cPAD generated in aqueous and nonpolar anti-solvents is related to the relative interaction propensity between the solvent, anti-solvent, and HPMCAS [60]. Similar to the relative interactions between API, polymers, and solvents that govern crystallization propensity from the supersaturated state [61,62,63], both solvent and anti-solvent interact with the co-precipitated amorphous dispersion when suspended in mother liquors. We hypothesize that preferential solvation by the aqueous anti-solvent can shield the dispersion from strongly plasticizing organic solvents and prevent crystallization. In contrast, nonpolar anti-solvents, such as *n*-heptane, are less able to shield the dispersion from organic solvent, explored in more detail below.

**Figure 6:**
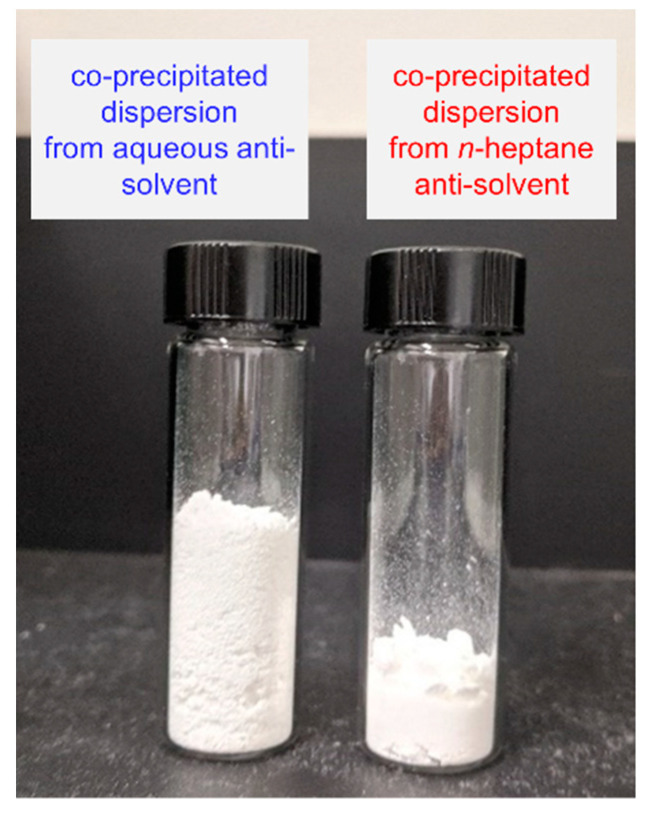
Equal amounts of material generated by precipitation into acidified water (0.18 g/cc bulk density) and *n*-heptane (0.3 g/cc).

Alongside crystallinity, the bulk powder properties of amorphous dispersions are crucial quality attributes impacting bioperformance [3]. Processing conditions to generate spray-dried or extruded amorphous dispersions can significantly impact powder properties, resulting in changes to the efficiency of downstream formulation, release kinetics of API, and tablet image size [13,19,35,64,65]. The powder properties for co-precipitated posaconazole dispersions, namely bulk density, were also found to depend on the anti-solvent used in the precipitation. Shown in Figure 6, co-precipitation from an aqueous anti-solvent resulted in a poor bulk density of the cPAD. This poor bulk density was observed regardless of solvent selection. In contrast, the co-precipitation into *n*-heptane produced a dispersion with improved bulk density, as well as qualitatively improved powder flow properties. Co-precipitation screens of internal compounds at the research laboratories of Merck & Co., Inc., Kenilworth, NJ, USA have found this relationship to be somewhat general; co-precipitation into aqueous anti-solvents tends to produce materials with a lower bulk density than precipitation into nonpolar anti-solvents. Although the bulk density of cPAD can be increased by processing alongside bulking excipients to form hierarchical particles [32,33,66], a high-bulk-density ASD containing solely API and a stabilizing polymer avoids the potentially complex phase behavior of hierarchical composite materials in the final formulation. Given the liabilities associated with residual crystallinity in the high-bulk-density posaconazole dispersion generated in *n*-heptane, we sought to identify a route to densify the amorphous dispersion generated from an aqueous anti-solvent without inducing the crystallization of Form III informed by the mechanisms of crystallization and densification during co-precipitation in *n*-heptane.

To understand the source of phase transformation for posaconazole, fully amorphous co-precipitated dispersions (precipitated from acetone into 0.001 N HCl) were suspended in various solvent combinations to identify which of these led to crystallization. These data are summarized in Table 2. Although dispersing amorphous cPAD in neat *n*-heptane did not result in crystallization, the addition of 10 vol% solubilizing solvents such as acetone or MEK resulted in Form III crystallization. Furthermore, suspending cPAD in heptane containing 10 wt% isopropanol, which does not dissolve HPMCAS, or suspending in methyl tert-butyl ether, which is a poor solubilizer of both the drug and polymer, induced crystallization of the amorphous dispersion. These data show that crystallization is highly sensitive to the presence of an organic solvent and is not necessarily a consequence of conditions during the co-precipitation event, but can also occur during the processing and storage of cPAD in a slurry. Additionally, there was no evidence of solubilization of posaconazole in these mother liquors by a gravimetric analysis. Co-precipitated dispersions washed with an excess of anti-solvent still showed conversion to Form III, suggesting that conversion occurred from the cPAD phase rather than during the evaporation of mother liquors during drying. Dispersing cPAD in acidified water containing 10 wt% acetone did not result in phase transformation of the amorphous dispersion, although the addition of immiscible MEK to the aqueous suspension resulted in crystallization. It was also observed that the powder properties of cPAD underwent transformation during suspension in a slurry. Summarized in Table 2, cPAD was densified when suspended in a non-polar anti-solvent containing organic solvent. As both crystallization and densification occurred concurrently for the amorphous cPAD, we hypothesized that both originate from solvent-induced plasticization of the ASD. In contrast, cPAD dispersed in aqueous anti-solvents was shielded from the impact of solvent, so long as that solvent is miscible with water. Solvent-induced plasticization and crystallization is a common failure mode during the processing of amorphous dispersions. The plasticization of spray-dried amorphous materials can lead to detrimental effects such as gumming and morphology collapse [67,68]; however, for co-precipitated dispersions, plasticization and deformation during slurry storage are favorable and densify the material to improve its downstream properties [16,69].

**Figure 7:**
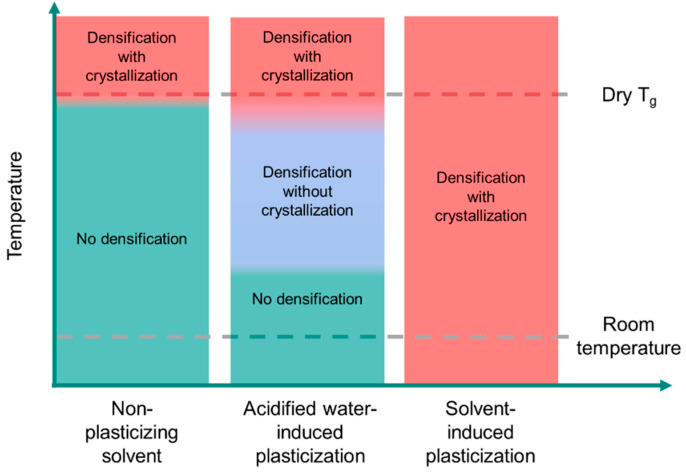
Conceptual schematic illustrating impact of temperature on densification and crystallization for posaconazole cPAD dispersed in non-plasticizing solvent (*n*-heptane), acidified water, and plasticizing organic solvents. The bounds on temperature for densification or crystallization depend on solvent or water content as well as the kinetics of annealing. Temperature axis is not shown to scale.

Processing conditions were screened to identify conditions to densify the cPAD cake without causing phase conversion. Observing the impact of solvent-induced plasticization on crystallization, as summarized above, temperature was employed rather than solvent to plasticize and densify the cPAD powder in its rubbery state. The T_g_ of dry posaconazole cPAD is just below 100 °C. Washing posaconazole cPAD with pure *n*-heptane heated to 100 °C produced a high-density powder, although the material showed form conversion to Form I by DSC. Although washing amorphous cPAD with room temperature 0.001 N HCl had no effect on the density of the precipitate, washing with 60 °C 0.001 N HCl produced a fully amorphous material of a high density. Water is a well-documented plasticizer of HPMCAS [70] and likely lowers the glass transition temperature of the amorphous dispersion to allow for densification at temperatures below the dry T_g_. However, unlike procedures that use solvent or more extreme heating to densify the cPAD, washing with hot water did not result in phase transformation to Form III. Posaconazole crystallization kinetics at 60 °C are sufficiently slow to prevent the formation of Form III in the dispersion, unlike during annealing at 100 °C. A conceptual schematic illustrating these results is shown in Figure 7. Nonpolar solvents do not significantly plasticize posaconazole cPAD, and at the temperatures required for densification, form conversion occurs to a crystalline phase. Acidified water is a moderate plasticizer of posaconazole cPAD; there is a window above the wetted T_g_ where phase conversion is slow and the amorphous dispersion can be densified with a hot water wash. The exact temperatures where densification and phase transformation occur depend on water content and the kinetics of annealing. For cPAD plasticized by organic solvents, cPAD undergoes densification and phase transformation to crystalline Form III across the entire temperature regime studied. Differences in morphology between the cPAD materials were also assessed by scanning electron microscopy (see Supporting Information). Upon densification, the low bulk-density cPAD powder collapses from a fibrous, porous microstructure to densified particles. T_g_ as a function of water or solvent content, as well as crystallization propensity for an amorphous dispersion, depends on the chemical structure of the dispersed API and composition of the solid state. For poorly water-soluble BCS II and IV compounds enabled by delivery from ASDs, plasticization by water is likely to be less significant than plasticization by polar organic solvents. Although a host of molecular properties determine physical stability risk during the processing of ASDs [71], we posit that washing with hot water may allow for densification without form conversion for many amorphous dispersions, allowing for improved bulk powder properties while retaining the solubility advantage of the amorphous phase.

Unlike spray drying or melt extrusion, particle formation and particle isolation during co-precipitation are decoupled events. As a result, co-precipitation requires isolation from mother liquors by filtration or evaporation and lends itself to a more diverse set of processing conditions during manufacturing than other routes, although there are growing examples of the post-processing of ASDs generated by spray drying or melt extrusion. High-density, fully amorphous dispersions of posaconazole can be achieved by leveraging particle formation in cold, aqueous anti-solvent and subsequent densification by washing with 60 °C 0.001 N HCl. It is crucial to precipitate the dispersion as a glass in a cold anti-solvent to prevent gumming and clogging in the wet mill used to generate and disperse the cPAD slurry, and controlling the temperature during densification is also critical to avoid crystallization. Yet, once formed, washing with hot water allows for densification without inducing crystallization. For posaconazole dispersions, such a route allows for the control over dispersion plasticity to provide high-bulk-density and fully amorphous solid dispersions of the compound.

## 4. Conclusions

Amorphous solid dispersions are enabling delivery vehicles to improve the oral bioavailability of an API for therapeutic efficacy [3]. However, solvent selection during the manufacturing of ASDs has existed as a primarily empirical exercise, yet the outcome of which can have a substantial impact on the molecular and material properties of dispersions. For co-precipitated amorphous dispersions of posaconazole, precipitation into aqueous anti-solvents was found to limit phase conversion by shielding the dispersion from the plasticizing impact of residual organic solvent. Furthermore, posaconazole cPAD can be densified to improve bulk powder properties by washing with hot aqueous anti-solvent. It is hypothesized that this mechanism proceeds via water-induced plasticization of the ASD, where T_g_ is reduced such that the densification of the rubbery dispersion occurs at temperatures where phase conversion is kinetically limited. Such a platform approach is applicable for a range of pharmaceuticals with relatively slow crystallization kinetics in their rubbery state, a common feature for a growing number of compounds with large molecular weight and functional group complexity [22,72]. We are confident that scientists in both industry and academia can adopt this processing approach to generate high-density ASDs to enable the successful production and formulation of low-solubility APIs in co-precipitated amorphous materials.

## Figures and Tables

**Table 1:** Impact of solvent selection on properties of co-precipitation dispersions of posaconazole in HPMCAS-M. Solvent:anti-solvent ratios of 1:10 were used for co-precipitation.

Anti-Solvent	Solvent	Posaconazole Phase
*n*-heptane	Acetone	Form III
	Acetone/water	Form III
	Methyl ethyl ketone	Form III
	Tetrahydrofuran	Form III
Cyclohexane	Methyl ethyl ketone	Form III
0.001 N HCl	Acetone	Amorphous
	Ethanol/water	Amorphous
	Tetrahydrofuran	Amorphous/amorphous phase separation

**Table 2:** Properties of amorphous cPAD precipitated and isolated from acetone/water and re-suspended in solvent combinations (10 vol% solvent relative to 90 vol% anti-solvent, unless otherwise listed). Solvent combinations that densified the cPAD without causing crystallization are in bold.

Anti-Solvent	Solvent	Posaconazole Phase	Bulk Density
*N*-heptane (5 °C)	None	Amorphous	Retained
*N*-heptane (5 °C)	Acetone	Form III	Densified
*N*-heptane (5 °C)	Methyl ethyl ketone	Form III	Densified
*N*-heptane (5 °C)	Isopropanol	Form III	Densified
*N*-heptane (100 °C)	None	Form I	Densified
Methyl tert butyl ether	None	Form III	Densified
0.001 N HCl (5 °C)	Acetone	Amorphous	Retained
0.001 N HCl (5 °C)	MEK	Form III	Densified
**0.001 N HCl (60 °C)**	**None**	**Amorphous**	**Densified**

## Data Availability

All data available are reported in the article.

## Supplementary Materials

The following are available online at https://www.mdpi.com/article/10.3390/pharmaceutics13122017/s1, Figure S1: mDSC of Form III posaconazole showing contamination by amorphous posaconazole, apparent by its Tg at 60 °C, Figure S2: Thermogram through Tg for posaconazole/HPMCAS dispersion generated by precipitation from MEK into n-heptane before (first cycle) and after (second cycle) heating to 150 °C, Figure S3: Posaconazole/HPMCAS co-precipitates generated using n-heptane as anti-solvent, Figure S4: Posaconazole/HPMCAS co-precipitate generated using cyclohexane as anti-solvent, Figure S5: Posaconazole/HPMCAS co-precipitates generated using 0.001 N HCl as anti-solvent, Figure S6: Amorphous posaconazole cPAD re-suspended in n-heptane and binary solvent mixtures, Figure S7: Amorphous posaconazole cPAD re-suspended in MTBE, Figure S8: Amorphous posaconazole cPAD re-suspended in 0.001 N HCl and binary solvent mixtures, Figure S9: Activity coefficients of acetone in binary solvent mixtures in a) water and b) n-heptane as predicted in Dynochem, Figure S10: Scanning electron microscopy of posaconazole cPAD generated by precipitation into 0.001 N HCl, Figure S11: Scanning electron microscopy of posaconazole cPAD generated by precipitation into n-heptane, Figure S12: Scanning electron microscopy of posaconazole cPAD generated by precipitation into 0.001 N HCl and washed with 60 °C acidified water.
