# Supplementary material for: Optimizing Solvent Selection and Processing Conditions to Generate High Bulk-Density, Co-Precipitated Amorphous Dispersions of Posaconazole
Source: Pharmaceutics. 2021 Nov 26;13(12):2017. doi: 10.3390/pharmaceutics13122017 (PMC8705469; doi:10.3390/pharmaceutics13122017)
Supplement: Supplementary file 1 [file pharmaceutics-13-02017-s001.zip › pharmaceutics-1456891-done.pdf]

pharmaceutics

MDPI

Supporting Information

# Optimizing Solvent Selection and Processing Conditions to Generate High Bulk-Density, Co-Precipitated Amorphous Dispersions of Posaconazole

Derek Frank[1,*], Luke Schenck[1], Athanas Koynov[1], Yongchao Su[2], Yongjun Li[2], Narayan Variankaval[2]

[1] Process Research & Development, Merck & Co., Inc, Rahway, NJ 07065, USA
[2] Analytical Research & Development, Merck & Co., Inc, Rahway, NJ 07065, USA
* Correspondence: derek.frank@merck.com

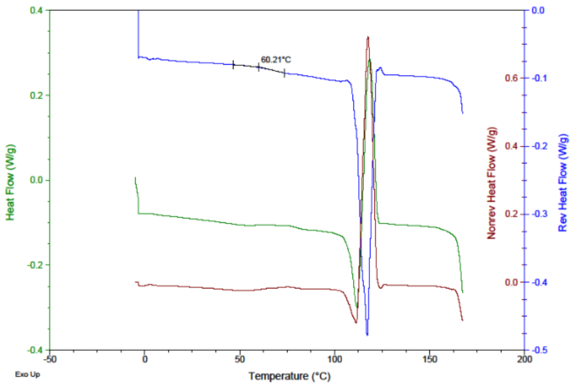


Figure S1. mDSC of Form III posaconazole showing contamination by amorphous posaconazole, apparent by its $T_g$ at 60 °C.

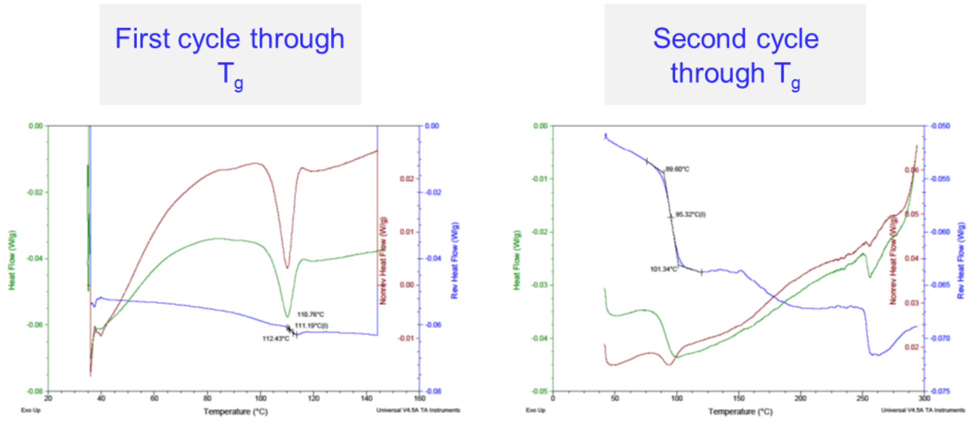


Figure S2. Thermogram through $T_g$ for posaconazole/HPMCAS dispersion generated by precipitation from MEK into *n*-heptane before (first cycle) and after (second cycle) heating to 150 °C.

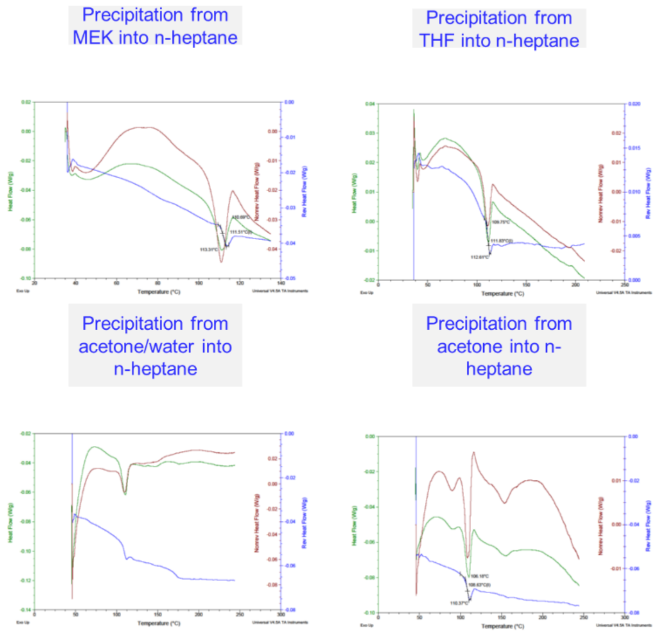


Figure S3. Posaconazole/HPMCAS co-precipitates generated using *n*-heptane as anti-solvent.

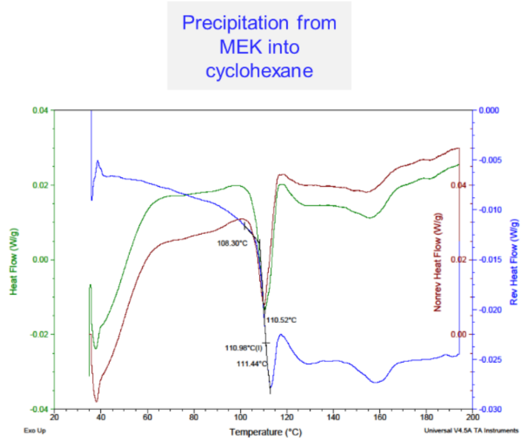


Figure S4. Posaconazole/HPMCAS co-precipitate generated using cyclohexane as anti-solvent.

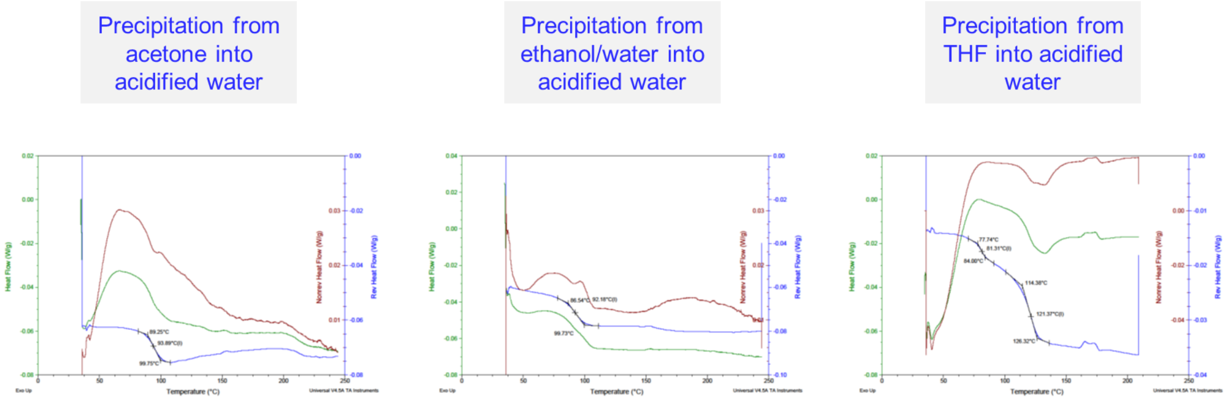


Figure S5. Posaconazole/HPMCAS co-precipitates generated using 0.001 N HCl as anti-solvent.

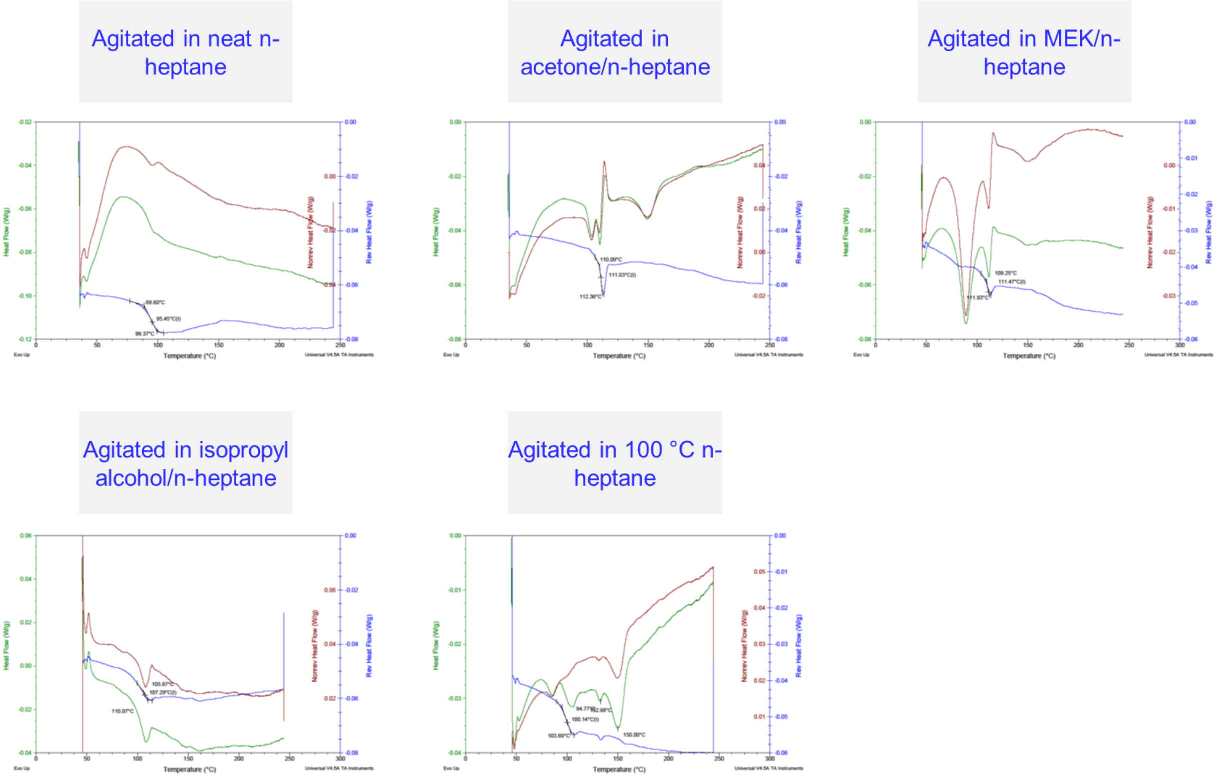


Figure S6. Amorphous posaconazole cPAD re-suspended in *n*-heptane and binary solvent mixtures.

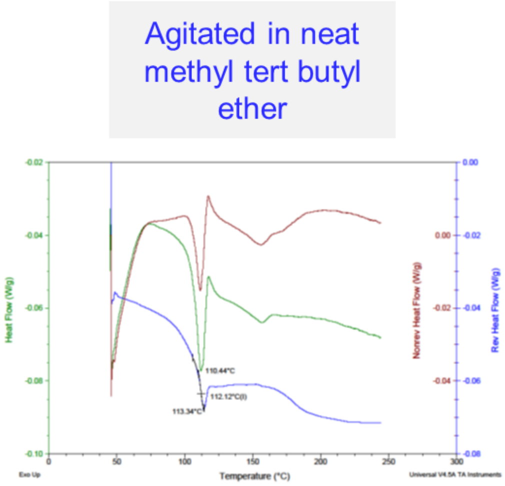


Figure S7. Amorphous posaconazole cPAD re-suspended in MTBE.

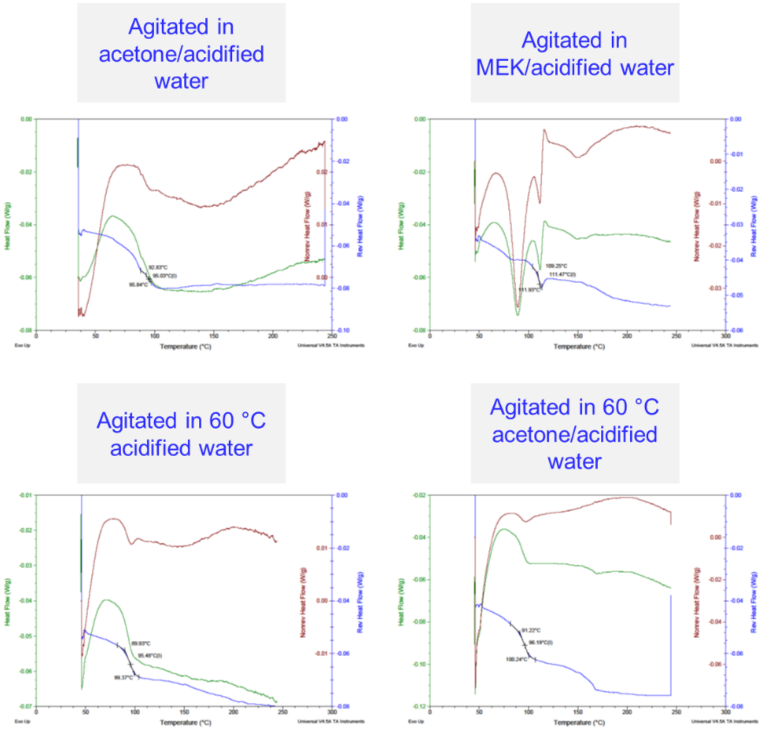


Figure S8. Amorphous posaconazole cPAD re-suspended in 0.001 N HCl and binary solvent mixtures.

*Dynochem prediction*

Activity coefficients of acetone in binary solvent mixtures in a) water and b) *n*-heptane as predicted in Dynochem using the NRTL feature.

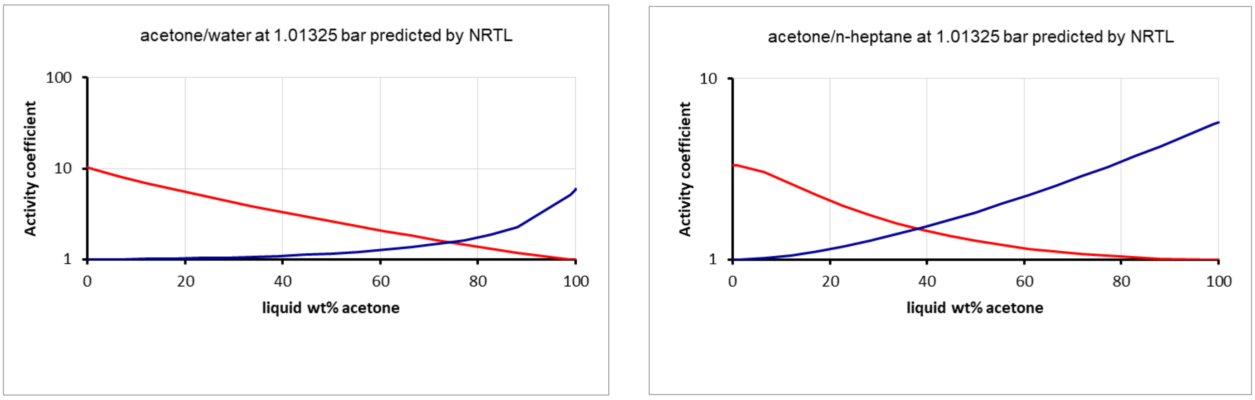


Figure S9. Activity coefficients of acetone in binary solvent mixtures in a) water and b) *n*-heptane as predicted in Dynochem.

*Scanning electron microscopy*

Particle morphology was assessed by scanning electron microscopy using a Tabletop Microscope TM3030 (Hitachi High-Technologies Co., Tokyo, Japan) at 15 kV acceleration voltage.

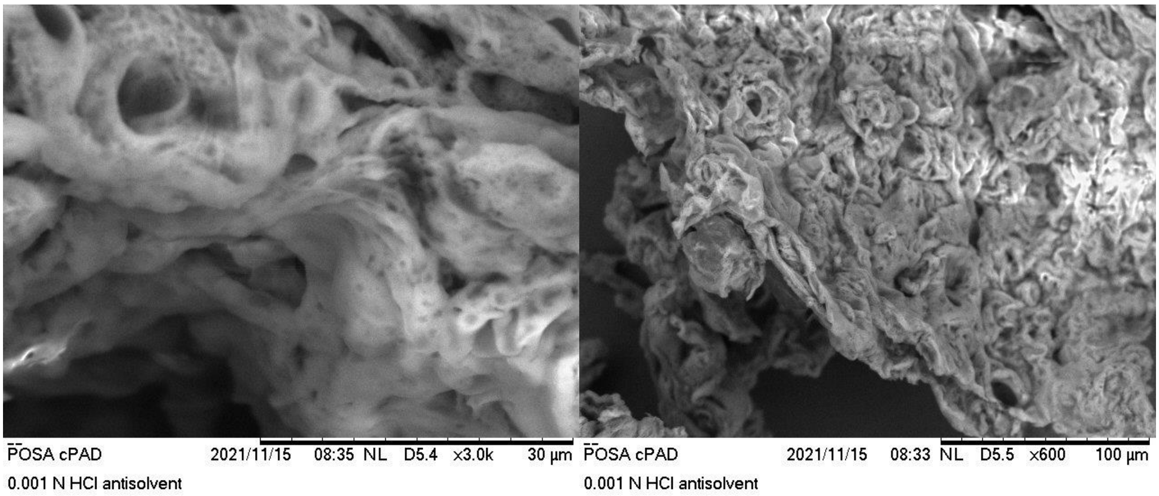


Figure S10. Scanning electron microscopy of posaconazole cPAD generated by precipitation into 0.001 N HCl.

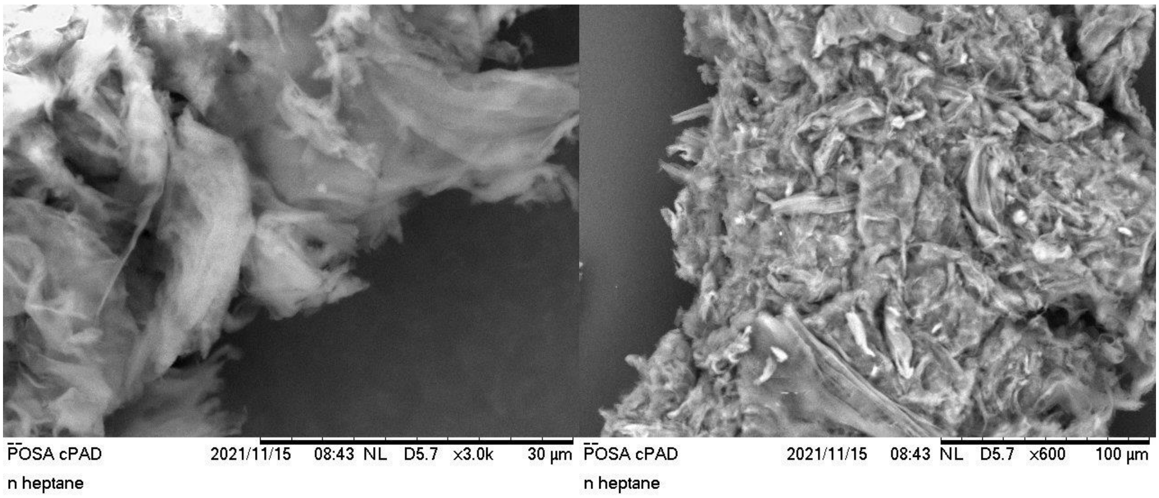


Figure S11. Scanning electron microscopy of posaconazole cPAD generated by precipitation into *n*-heptane.

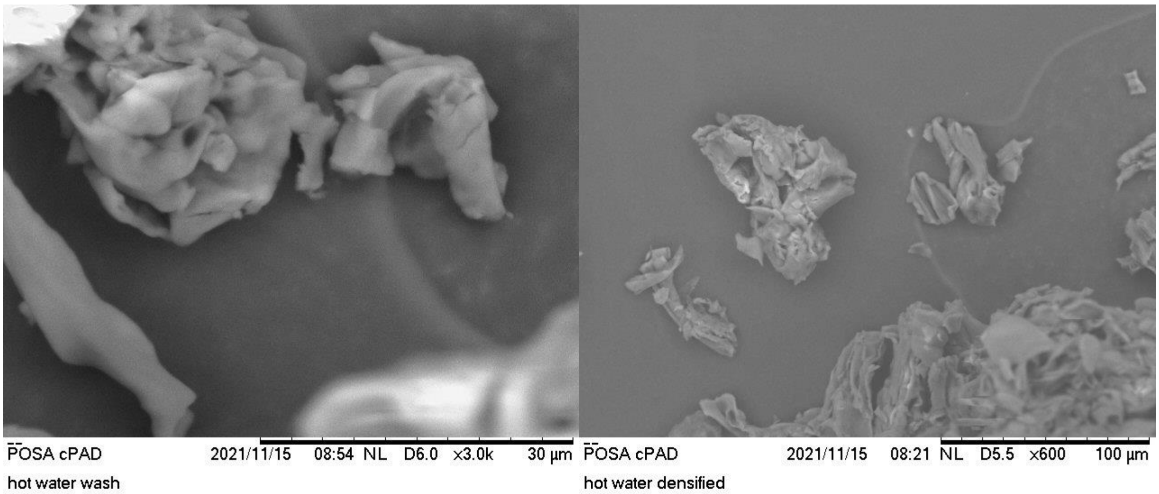


Figure S12. Scanning electron microscopy of posaconazole cPAD generated by precipitation into 0.001 N HCl and washed with 60 °C acidified water.
